# UK General Population Utility Values for the SIDECAR-D Instrument Measuring the Impact of Caring for People With Dementia

**DOI:** 10.1016/j.jval.2020.04.1827

**Published:** 2020-08

**Authors:** Edward J.D. Webb, David Meads, Hareth Al-Janabi, Paul Kind, Francesca Torelli, Mike Horton, Jan Oyebode, Penny Wright

**Affiliations:** 1 Leeds Institute of Health Sciences, University of Leeds, Leeds, England, UK; 2 Institute of Applied Health Research, University of Birmingham, Birmingham, England, UK; 3 Leeds Institute of Rheumatic and Musculoskeletal Medicine, University of Leeds, Leeds, England, UK; 4 Centre for Applied Dementia Studies, University of Bradford, Bradford, England, UK; 5 Leeds Institute of Medical Research at St James’s, University of Leeds, Leeds, England, UK

**Keywords:** carers, dementia, quality of life, valuation, best-worst scaling, visual analog scale

## Abstract

**Objectives:**

Dementia affects many people, with numbers expected to grow as populations age. Many people with dementia receive informal/family/unpaid care, for example, from a spouse or child, which may affect carer quality of life. Measuring the effectiveness of health/social care interventions for carers requires a value measure of the quality-of-life impact of caring. This motivated development of the Scales Measuring the Impact of Dementia on Carers-D (SIDECAR-D) instrument. This study aimed to obtain general population values for SIDECAR-D to aid incorporating the impact of caring in economic evaluation.

**Methods:**

Members of the UK general public completed a best–worst scaling object case survey, which included the 18 SIDECAR-D items and EQ-5D-3L descriptions. Responses were analyzed using scale-adjusted finite mixture models. Relative importance scores (RISs) for the 18 SIDECAR-D items formed the SIDECAR-D relative scale measuring the relative impact of caring. The SIDECAR-D tariff, on the full health = 1, dead = 0 scale, was derived by rescaling EQ-5D-3L and SIDECAR-D RISs so the EQ-5D-3L RISs equaled anchored valuations of the EQ-5D-3L pits state from a visual analog scale task.

**Results:**

Five hundred ten respondents completed the survey. The model had 2 parameter and 3 scale classes. Additive utility decrements of SIDECAR-D items ranged from –0.05 to –0.162. Utility scores range from 0.95 for someone affirming 1 item to –0.297 for someone affirming all 18.

**Conclusion:**

SIDECAR-D is a needs-based scale of the impact on quality of life of caring for someone with dementia, with a valuation tariff to support its use in economic evaluation.

## Highlights

• Many people with dementia receive informal care; Scales Measuring the Impact of Dementia on Carers-D (SIDECAR-D) is a survey instrument measuring the burden of caring.
• Two scoring systems are provided, the SIDECAR-D relative scale, which measures the relative burden of caring on a 0-100 scale, and the SIDECAR-D tariff, which measures the burden on a scale anchored at full health = 1 and dead = 0.
• Utility decrements of SIDECAR-D items can be as great as –0.162 on the full health = 1, dead = 0 scale.

## Introduction

Dementia is a syndrome linked to a range of progressive brain diseases causing gradual decline in mental abilities and increasing functional difficulties. People with dementia often have care provided by family or friends. Being a caregiver can place a large burden on people in time and resources, disruption to daily life, and psychological effects.^1^

Dementia mainly affects those aged over 65, and increased life expectancy means that globally the number of people diagnosed is expected to rise from 47 million in 2015 to 3 times that by 2050.^2^ As the number of people with dementia increases, so too does the societal burden, meaning greater need for support and services targeting people with dementia and their carers.

By *carer* we refer to any person providing care for someone with dementia who is not formally employed to do so. This definition encompasses the terms *informal carer*, *family carer*, and *unpaid carer* used elsewhere.

Efficient and optimal allocation of resources in this area requires preference-based quality-of-life measures, for both people with dementia and their carers. The relationship between severity of dementia and impact on a carer’s quality of life is nonlinear. For example, a worsening of the condition may lead to greater provision of formal support, or the person moving into a nursing home, reducing carer burden. Carers may also adjust to their caring role over time, meaning their quality of life improves as severity of dementia increases.^3^ Thus, extrapolating the impact on carers from knowledge about people with dementia is impossible: carers’ quality of life must be measured directly.

Assigning values to the impact of caring allows the comparison of different individuals, different points for individuals, and the effectiveness of carer-targeted interventions. In addition, interventions targeted at people with dementia themselves may improve quality of life for their carers, termed *spillovers*. Recent National Institute for Health and Care Excellence and US panel guidance^4^^,^^5^ on cost-effectiveness highlights the need to develop methods for valuing spillovers to formally consider them within economic evaluation, to determine the true overall benefit of interventions, and make decisions that maximize benefits from scarce healthcare resources.^6^

In many health systems, including the UK National Health Service,^7^ the preferred measure of health benefit is quality-adjusted life-years (QALYs), a composite endpoint comprising both quality and length of life. Quality is captured in utility or preferences for health states where 1 is defined as full health, 0 is defined as equivalent to dead, and negative values represent health considered worse than dead. Thus, 1 QALY is the equivalent of living 1 year in full health.

Because caring for someone with dementia can have an impact on all aspects of an individual’s life, generic health measures such as EQ-5D may detect certain aspects of health-related caring impact, for example, preventing people from performing their usual activities. Health-related quality-of-life measures have been used with family carers in economic evaluations, including in dementia.^8^ Nevertheless, there is concern they capture only limited aspects of the impact of caring on quality of life.^9^^,^^10^ Measures including care-related items, such as relationships, fulfillment, and support, might be more appropriate for capturing carer quality of life. Many such general measures exist,^11^^,^^12^ but few have preference-based scoring algorithms for use in economic evaluation.

The Adult Carers Quality of Life questionnaire^13^ has been successfully employed in assessing carer services,^14^ including for carers of people with dementia.^15^ Nevertheless, Adult Carers Quality of Life questionnaire items have not been valued, meaning the relative impact of each cannot be assessed. The CarerQoL^16^ and Carer Experience Scale (CES)^17^ have been valued using choice-based methods. Nevertheless, the outcome scores cannot be obviously compared to, or summed with, patient QALYs, because they are not on the full health = 1, dead = 0 scale. The Dementia Quality of Life Scale for Older Family Carers^18^ is specific to dementia carers, but only a subset (older family members). This neglects many younger carers, for example, those caring for a parent, for a spouse with young-onset dementia, or for friends. Valuation is also not available for the Dementia Quality of Life Scale for Older Family Carers.

There is hence a need for a scale assessing the impact on quality of life of caring for someone with dementia that reflects their experiences, summarizes the relative severity of the quality-of-life impact, and generates QALY values for economic assessment. The DEmentia Carers Instrument DEvelopment project created the SIDECAR (Scales measuring the Impact of DEmentia on CARers) instrument. SIDECAR was developed using a needs-led approach, with items generated from carers only, using exact phrases where possible. Development and evaluation followed Consensus-Based Standards for the Selection of Health Measurement Instruments,^19^ including interviews (N = 42),^20^^,^^21^ to generate an item pool and psychometric evaluation of the initial item pool.^22^ This resulted in 3 instruments, SIDECAR-D (18 items), which measures the direct impact of caring for someone with dementia; SIDECAR-I (10 items), which measures the indirect effect; and SIDECAR-S (11 items), which measures support and informational needs.^22^

This article details a valuation study of SIDECAR-D. Two measures are presented, the SIDECAR-D relative scale (SIDECAR-D-RS), which measures the relative impact of caring for someone with dementia on a 0 to 100 scale, and the SIDECAR-D tariff, which gives the impact on a scale with full health = 1 and dead = 0. The SIDECAR-D-RS is an easy-to-understand relative scale that can be used to compare groups of carers or one group over time. The SIDECAR-D tariff, being anchored to the scale usually used in analyzing health and care interventions, may be used to compare carer interventions with interventions in other fields, and to evaluate carer spillovers. Different ranges for the measures were deliberately chosen to emphasize that they are on different scales.

## Methods

### Survey Development

SIDECAR-D items are scored using a binary agree/disagree response format. The time frame reference is “today.” Examples are “I don’t take very good care of myself” and “I often feel I want to escape my caring responsibilities.” The full list of items is not publicly available, although SIDECAR is free for use in public health, social care, voluntary sector, and not-for-profit organizations following registration (to use SIDECAR, please register with the University of Leeds Fast Licensing Platform at www.licensing.leeds.ac.uk).

The valuation exercise used best-worst scaling object case (BWS-OC) (also known as BWS case 1). It elicits relative preference for many items by presenting a small subset, using the simple task of survey participants choosing which item is best (or alternative term depending on context) and which is worst. Although the related method of BWS profile case has been used for valuation^17^^,^^23^ and some BWS-OC studies in healthcare exist,^24^ we are not aware of a study using BWS-OC to value a survey instrument.

Studies valuing items on a full health = 1, dead = 0 scale often use time-trade off (TTO)^25^ or standard gamble (SG).^26^ Best–worst scaling object case was carefully chosen to suit SIDECAR-D. In TTO and SG, survey participants are shown health states or vignettes constructed from the items being valued. In the former, they identify the length of life in full health that they consider equivalent to 10 years in the state being valued. In the latter, they state the highest probability of instant death they would risk to live 10 years in full health rather than live 10 years in the state being valued.

Both methods were unsuitable here. Completing SIDECAR-D entails asking individuals to agree or disagree with a list of statements, making it difficult to create descriptions of states without using negations of the statements that do not form part of the instrument. It would be possible to create vignettes by listing the statements agreed with, but there are 18 items, some comprising lengthy sentences originating in voices of carers themselves, reflecting the realities and nuances of their experiences. Thus vignettes for severe SIDECAR-D states would be overly long and difficult for respondents to take in. In addition, the trade-off between quality and length of life is not as intuitive for carers as it is for patients, because their own condition is not being valued. Caring for someone means that to some extent someone is dependent on them, which may also influence their willingness to trade the possibility of death. Finally, there are 2^18^ = 262 144 SIDECAR-D states, making valuing any meaningful subset of them using TTO or SG impractical.

Another alternative is discrete choice experiments (DCEs),^27^ in which individuals choose which of 2 options they prefer. Discrete choice experiments can value a large number of states, and there is some evidence that they are more reliable than BWS profile case.^28^ Although they could in theory be employed here, choosing between 2 potentially lengthy SIDECAR-D vignettes would present an impractical burden for survey participants.

The five EQ-5D level 3 descriptions were added to the 18 SIDECAR-D items and a statistical design constructed using the crossdes package for R. A design was created that balanced in order of priority (1) items, (2) pairs of items, and (3) items in a given position appearing an equal amount of times. The design had 6 versions, each with 8 questions. Participants were shown 6 items and indicated which would have the most negative and least negative impact on their quality of life. For an example, see the survey instrument which is included as Supplemental Materials found at https://doi.org/10.1016/j.jval.2020.04.1827.

Participants also completed 2 visual analog scale (VAS) tasks, rating health states on a scale from 100 (the best health you can imagine) to 0 (the worst health you can imagine). A screenshot is included in the survey instrument provided as Supplemental Materials found at https://doi.org/10.1016/j.jval.2020.04.1827. Participants rated their own health today, then rated 11111, 33333, and dead. From this task a valuation of 33333 on the full health = 1, dead = 0 scale was obtained and used to anchor valuations. Finally, participants answered questions about themselves (age, sex, etc.) to assess the sample’s representativeness. For details, see the survey instrument provided as Supplemental Materials found at https://doi.org/10.1016/j.jval.2020.04.1827.

The survey was tested with 10 carers, including carers for people with dementia and wording of questions and instructions refined in response to feedback. The survey was administered online, through a survey company, and collected a representative UK general public sample from an existing panel. The survey was piloted with 50 respondents. Responses were gathered between August 22, 2018, and September 2, 2018, with a recruitment target of 500 (including 50 from the pilot, because no further changes were made). Formal power calculations were not performed; however, the sample size was considered more than adequate to estimate robust statistical models based on past experience and previous literature.^24^

### Analysis

The decision utility to a participant of selecting item i as having the most negative impact on quality of life is modeled as being $u_{i}=V_{i}+\varepsilon_{i}$ where

$$V_{i}=\sum_{a=1}^{23}\beta_{a}x_{ia}$$

with $\beta_{a}$a vector of coefficients, $x_{ia}$a vector of dummy variables indicating whether item i contains attributea, and $\varepsilon_{i}$ an independent and identically distributed error term following a Gumbel distribution. The decision utility of selecting itemj as the least negative impact is $u_{j}=-\left(V_{j}+\varepsilon_{j}\right)$. Note that decision utilities are on a different scale than quality-of-life utility because it is convenient to model individuals receiving positive decision utility payoffs for identifying the most negative quality-of-life impact. It is assumed that individuals first select the item having the most negative impact, then the item having the least negative impact. The probability of a given choice is then

$$P\left(i\;most\;negative,\;j\;least\;negative\right)=\left(\frac{e^{\sigma V_{i}}}{\sum_{k}e^{\sigma V_{k}}}\right)\left(\frac{e^{-\sigma V_{j}}}{\sum_{k\neq i}e^{-\sigma V_{k}}}\right).$$

Where σ≥0 is the response scale indicating how much responses are explained by the deterministic part of the model and how much by the random part.^29^

Coefficients were transformed to relative importance scores (RISs) using Orme’s method.^30^ Following Zhang et al,^31^ the link between the relative importance, $I_{a}$, of an attribute in BWS-OC and level coefficients is modeled as

$$\alpha I_{a}=\left(\beta_{a}^{max}-\beta_{a}^{min}\right)$$

where $\beta_{a}^{max}$ and $\beta_{a}^{min}$ are respectively the largest and smallest coefficients associated with attribute a. Here, $\beta_{a}^{max}$ is the level 1 EQ-5D coefficient, by definition 0, and $\beta_{a}^{min}$ the level 3 utility decrement on a given EQ-5D dimension. Summing across all EQ-5D dimensions:

$$\sum_{a=1}^{5}I_{a}^{EQ-5D}=\sum_{a=1}^{5}\beta_{a}^{level\;3}$$

VAS valuations of state 33333 on the full health = 1, dead = 0 scale are found from

$$VAS_{33333}^{0\;-\;1}=\frac{VAS_{33333}-VAS_{dead}}{VAS_{11111}-VAS_{dead}}.$$

By equating $\sum_{a=1}^{5}\beta_{a}^{level\;3}$ and $VAS_{33333}^{0\;-\;1}$, RIS from the BWS-OC are anchored to the full health = 1, dead = 0 scale by multiplying by

$$\alpha =\frac{1-\;VAS_{33333}^{0-1}}{\sum_{a=1}^{5}I_{a}^{EQ-5D}}.$$

The anchoring approach described above requires individuals to give logically consistent VAS responses, that is, $VAS_{11111}>VAS_{33333}$ and $VAS_{11111}>VAS_{dead}$ (though it does not preclude $VAS_{dead}>VAS_{33333}$, that is, 33333 considered worse than dead). Thus respondents giving illogical responses were excluded from analysis.

Scale-adjusted finite mixture models^32^ were estimated, which allow for $n_{P}$ classes of response parameters and $n_{S}$ scale parameters, with the first normalized to 1. The probability of belonging to a given parameter (scale) class p (s) is $e^{\theta_{p}}/\sum_{i=1}^{n_{P}}e^{\theta_{i}}$ $\left(e^{\phi_{s}}/\sum_{i=1}^{n_{S}}e^{\phi_{i}}\right)$ with the θs and ϕs parameters to estimate and $\theta_{1}=\phi_{1}=0$.

Sixteen models were estimated with between 1 and 4 preference and between 1 and 4 scale classes. The final preferred model was the one minimizing the Bayesian Information Criterion.

The SIDECAR-D tariff, anchored to the full health = 1, dead = 0 scale, is calculated by taking the mean anchored valuations for each parameter and scale class weighted by probability of class membership. Similarly, the SIDECAR-D relative scale, giving the relative impact on an individual on a 0 to 100 scale, is found by taking a weighted mean over classes of RIS regarding only SIDECAR-D items. Note that SIDECAR-D-RS and the SIDECAR-D tariff have different endpoints as well as moving in opposite directions: a high relative scale score implies a large quality-of-life impact, whereas a high tariff value implies a low impact.

Statistical significance was judged at the 5% level after adjustment using Holm’s sequential Bonferroni correction.^33^ Design and analysis was conducted using R version 3.3.1, with models estimated using the Choice Modelling Centre Code for R version 1.1.^34^

## Results

Five hundred ten respondents completed the survey, of which 38 (7.45%) were excluded for illogical VAS responses, leaving 472 for analysis.

Table 1 summarizes respondent demographics. Women are overrepresented both in the full (58.6%) and analysis (59.7%) sample compared to the UK population (50.6%),^35^ with more men excluded for illogical VAS responses. The full and analysis samples are similar in age, education, and occupation. Respondents generally reported good health, with 44.9% of the analysis sample reporting being in state 11111. The proportion is somewhat lower (39.5%) for respondents who gave illogical VAS responses, but the full sample is similar (44.5%) to the analysis sample.

**Table 1:** Respondent demographics and summary of VAS responses.

	All respondents	Illogical VAS	Analysis sample
Sex (%)			
Female	58.6	44.7	59.7
Male	41	52.6	40
Other/prefer not to say	0.392	2.63	0.212
Age			
Mean	48.4	45.2	48.7
SD	(14.7)	(14)	(14.7)
Education (%)			
Left school after min age	76.9	68.4	77.5
Degree	56.7	63.2	56.1
Occupation (%)			
(Self-)employed	58.8	71.1	57.8
Retired	20.4	13.2	21
Housework	8.63	2.63	9.11
Student	2.16	5.26	1.91
Unemployed	3.92	2.63	4.03
Other	6.08	5.26	6.14
Self-report 11111 (%)	44.5	39.5	44.9
VAS 11111			
Mean	88.4	51.4	91.4
SD	(19.3)	(32.3)	(14.2)
VAS 33333			
Mean	20.3	51.5	17.8
SD	(20.3)	(32.6)	(16.6)
VAS dead			
Mean	11.4	62.2	7.34
SD	(22.2)	(37.8)	(14)
VAS own health			
Mean	73.7	66.3	74.3
SD	(19.4)	(21.1)	(19.1)
VAS 33333 (anchored)			
Mean	-	-	0.0278
SD	-	-	(1.06)
N	510	38	472

SD indicates standard deviation; VAS, visual analog scale.

Mean VAS ratings in the analysis sample are plausible, with 91.4 for 11111, 17.8 for 33333, and 7.34 for dead, and a mean anchored valuation of 33333 of 0.03. Respondents giving illogical VAS responses have less plausible ratings, with 51.4, 51.5, and 62.2, respectively.

Table 2 summarizes the analysis sample’s BWS-OC choices. Five of the top 6 differences in the most negative and the least negative responses are for EQ-5D items. These were chosen as having the most negative impact on quality of life far more often than they were chosen as having the least negative impact. At the other end of the scale, several items were chosen as having the least negative impact far more often than the most negative impact. This variation is an indication participants understood the tasks and could make meaningful distinctions between the impacts of different items.

**Table 2:** Best-worst scaling responses summary.

Attribute	Times presented	Most negative	Least negative	Difference
EQ-5D pain/discomfort L3	1023	526	33	493
EQ-5D mobility L3	1020	444	46	398
EQ-5D anxiety/depression L3	1009	355	46	309
EQ-5D self-care L3	956	276	40	236
SIDECAR-A item 5	961	260	109	151
EQ-5D usual activities L3	1010	187	106	81
SIDECAR-A item 14	945	159	79	80
SIDECAR-A item 1	1028	168	97	71
SIDECAR-A item 12	958	143	113	30
SIDECAR-A item 4	1027	144	119	25
SIDECAR-A item 13	934	112	117	−5
SIDECAR-A item 6	1013	128	149	−21
SIDECAR-A item 7	941	122	199	−77
SIDECAR-A item 3	1028	106	235	−129
SIDECAR-A item 10	1007	89	218	−129
SIDECAR-A item 18	953	52	186	−134
SIDECAR-A item 8	943	62	196	−134
SIDECAR-A item 11	937	53	194	−141
SIDECAR-A item 2	1015	88	239	−151
SIDECAR-A item 9	948	81	275	−194
SIDECAR-A item 16	1016	66	276	−210
SIDECAR-A item 15	954	61	330	−269
SIDECAR-A item 17	1030	94	374	−280

SIDECAR-A indicates Scales Measuring the Impact of Dementia on Carers-A.

Table 3 gives models’ Bayesian Information Criterion. The optimal fit had 2 preference and 3 scale classes. Table 4 gives the model coefficients. The second scale class has the largest scale parameter, that is, lowest error variance (14.6), followed by the third (7.61), then the first (normalized to 1). Respondents were most likely to belong to the second class (probability = 0.44), then the first (probability = 0.31), then the third (probability = 0.25). There are differences between the 2 parameter classes. In particular, in class 2 the EQ-5D items have the 5 largest coefficients, whereas in class 1, there is a SIDECAR-D item in the top 5, and usual activities has only the tenth highest coefficient. Respondents were most likely to belong to parameter class 2 (probability = 0.59).

**Table 3:** Bayesian information criterion for scale-adjusted finite mixture models.

	1 scale class	2 scale classes	3 scale classes	4 scale classes
1 parameter class	22930.45	22022.22	21983.44	21999.91
2 parameter classes	22150.9	21921.31	21910.66∗	21926.69
3 parameter classes	22348.57	21961.02	21992.62	22046.5
4 parameter classes	22176.33	22183.62	22100.93	22037.96

∗ Lowest value.

**Table 4:** Estimated parameters from final scale-adjusted finite mixture model.

Attribute	Parameter class 1	SE	Parameter class 2	SE
SIDECAR-A item 1	0.0194	(0.115)	0.113	(0.0521)
SIDECAR-A item 2	−0.0513	(0.112)	−0.218	(0.0576)
SIDECAR-A item 3	−0.0108	(0.0630)	−0.304	(0.0586)
SIDECAR-A item 4	−0.0021	(0.131)	−0.0006	(0.00900)
SIDECAR-A item 5	0.189	(0.142)	−0.0197	(0.0575)
SIDECAR-A item 6	−0.0669	(0.140)	0.0215	(0.0619)
SIDECAR-A item 7	−0.0515	(0.131)	−0.144	(0.0565)
SIDECAR-A item 8	−0.0572	(0.122)	−0.199	(0.0543)
SIDECAR-A item 9	−0.0908	(0.106)	−0.295	(0.0623)
SIDECAR-A item 10	−0.0746	(0.115)	−0.217	(0.0478)
SIDECAR-A item 11	−0.0543	(0.119)	−0.206	(0.0545)
SIDECAR-A item 12	0.0395	(0.121)	0.0479	(0.0571)
SIDECAR-A item 13	0.011	(0.126)	−0.0779	(0.0477)
SIDECAR-A item 14	0.0727	(0.122)	0.0769	(0.0517)
SIDECAR-A item 15	−0.0915	(0.117)	−0.415	(0.0725)
SIDECAR-A item 16	−0.084	(0.119)	−0.275	(0.0597)
SIDECAR-A item 17	−0.171	(0.150)	−0.306	(0.0620)
SIDECAR-A item 18	−0.0801	(0.115)	−0.162	(0.0521)
EQ-5D mobility L3	0.132	(0.140)	0.682	(0.112)
EQ-5D self-care L3	0.0742	(0.122)	0.476	(0.0872)
EQ-5D usual activities L3	0.0067	(0.122)	0.264	(0.0636)
EQ-5D pain/discomfort L3	0.174	(0.170)	0.748	(0.119)
EQ-5D anxiety/depression L3	0.167	(0.160)	0.41	(0.0810)
$\theta_{c}$	0	–	0.354	(0.238)

	Response scale	SE	$\phi_{s}$	SE
Scale class 1	1	–	0	–
Scale class 2	14.6	(2.79)	0.372	(0.322)
Scale class 3	7.61	(1.57)	−0.194	(0.366)

SE indicates standard error; SIDECAR A, Scales Measuring the Impact of Dementia on Carers-A.

**Figure 1:**
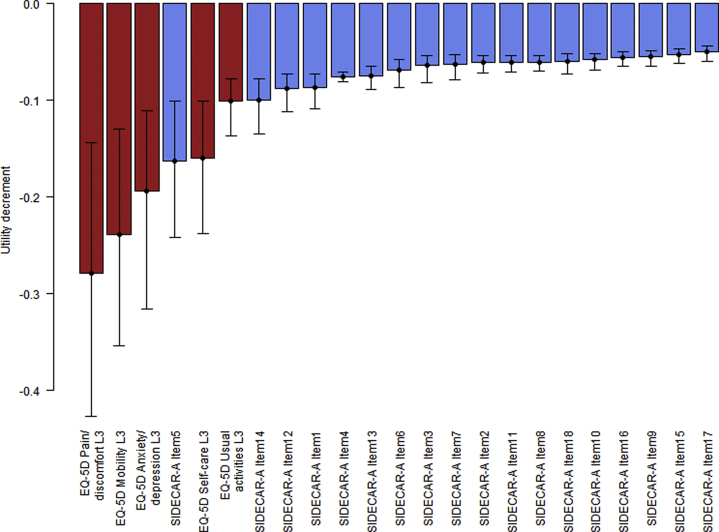
Utility decrements for Scales Measuring the Impact of Dementia on Carers-A (SIDECAR-A) items and EQ-5D-3L descriptions. Error bars show 95% confidence intervals.

Table 5 shows the SIDECAR-D relative scale and tariff, with Figure 1 showing utility decrements for SIDECAR-D items and EQ-5D level 3 descriptions. The largest utility decrement for an item is –0.162 on the SIDECAR-D tariff, equivalent to 15.2 on the SIDECAR-D-RS. All other items lower utility by 0.1 or less on the SIDECAR-D tariff. Scores on the SIDECAR-D tariff range from 0.95 if affirming only item 17 to –0.297 if affirming all 18 items.

**Table 5:** SIDECAR-A tariff and SIDECAR-A-RS.

	SIDECAR-A tariff	SIDECAR-A-RS
SIDECAR-A item1	−0.087	9.44
SIDECAR-A item2	−0.061	3.71
SIDECAR-A item3	−0.064	3.77
SIDECAR-A item4	−0.076	6.55
SIDECAR-A item5	−0.162	15.2
SIDECAR-A item6	−0.069	6.34
SIDECAR-A item7	−0.063	4.18
SIDECAR-A item8	−0.060	3.76
SIDECAR-A item9	−0.055	3
SIDECAR-A item10	−0.058	3.51
SIDECAR-A item11	−0.061	3.76
SIDECAR-A item12	−0.088	8.58
SIDECAR-A item13	−0.074	5.72
SIDECAR-A item14	−0.100	10.1
SIDECAR-A item15	−0.053	2.62
SIDECAR-A item16	−0.056	3.11
SIDECAR-A item17	−0.050	2.64
SIDECAR-A item18	−0.060	3.99
Example calculation:	Respondent affirms items 2, 4, 11, and 16 SIDECAR-A tariff score: 1 – 0.061 – 0.076 – 0.061 – 0.056 = 0.746 SIDECAR-A-RS score: 3.71 + 6.55 + 3.76 + 3.11 = 17.13	

SIDECAR-A indicates Scales Measuring the Impact of Dementia on Carers-A; SIDECAR-A

RS, Scales Measuring the Impact of Dementia on Carers Relative Scale.

## Discussion

This study successfully assigned values to SIDECAR-D items. Values of some items on the full health = 1, dead = 0 scale are considerable. For example, item 5, “I dread the future,” has a value of -0.162, a greater impact than EQ-5D-3L level 3 descriptions for self-care and usual activities. Even SIDECAR-D items of lower magnitude are comparable to level 2 coefficients from the EQ-5D five-level value set for England.^36^ Previous exercises valuing CES and the ICEpop CAPability measure (ICECAP), both aimed at a population with some overlap with this one, also found large utility decrements.^17^^,^^23^^,^^37^ Nevertheless, CES/ICECAP define the lowest state in the descriptive system as 0, thus values are not comparable, and as decrements must sum to -1, large decrements are found by construction.

This highlights that the UK general population recognize that caring for someone with dementia can have a large impact on the carer’s life. Items generated from the voices of carers resonate with and are understood by the general public, reaffirming the value of a bottom-up, carer-led process in developing a measure.

Comparing the conceptual nature of BWS-OC to alternatives, TTO may be thought of as giving a valuation for a particular state, whereas DCE responses can be analyzed to provide a valuation of a descriptive system, both from the perspective of an average member of the population. BWS-OC responses when analyzed give the relative importance of a set of items, again from the average population perspective.

Comparing what these values mean, BWS-OC is sometimes used solely as a user-friendly alternative to ranking items, or as a replacement for a VAS/rating scale.^38^ Nevertheless, it is possible to extract much more information from responses, because BWS-OC can be grounded in random utility theory,^39^ in the same way as DCEs. Conceptually, this means participants’ responses to BWS-OC tasks reflect the same underlying utility scale as DCE, TTO, and SG tasks. Empirical evidence supports BWS-OC tasks measuring the same utilities as DCEs (up to a scale transformation).^31^^,^^40^ Values from BWS-OC are thus a utility-based measure, satisfying the National Institute for Health and Care Excellence’s requirements for measures suitable for use in health technology assessment.^4^

This study compares the specific impact on carers, represented by SIDECAR-D, and general health impacts, represented by the EQ-5D. The conceptual validity of doing so rests on both reflecting the same underlying latent utility scale. SIDECAR-D hence contrasts with, for example, ICECAP, which seeks to measure capability.^41^ Previous studies have illustrated the feasibility of relating condition-specific measures and the EQ-5D. An example is exercises mapping the cancer-specific measures European Organisation for Research and Treatment of Cancer (EORTC) and Functional Assessment of Cancer Therapy-General (FACT-G) to the EQ-5D.^42^, ^43^, ^44^ Another example is bolt-ons to EQ-5D, which add another, condition specific, dimension, for example, vision.^45^ In exercises valuing bolt-ons, survey participants are presented with combinations of EQ-5D and specific items, analogous to the exercise presented here.

Quality-adjusted life-years calculated using the SIDECAR-D tariff are potentially very different from those generated from health-related instruments such as the EQ-5D, yet both are on the same scale. Thus, in principle at least, interventions targeted at carers for people with dementia or spillovers from interventions for people with dementia may be directly and meaningfully compared to health-focused interventions. This is potentially of great benefit, especially given recognition of the importance of addressing the needs of a household or family to sustain caring for people with dementia in the community. There are also movements toward greater integration of health and social care,^46^^,^^47^ implying a greater need to compare disparate interventions to optimally allocate resources. It is hoped measures such as SIDECAR-D can aid such comparisons.

There were several limitations to this study. Some SIDECAR-D items may capture some of the essence of EQ-5D descriptions using more vivid language. For example, item 15, “caring prevents me from fulfilling my other activities,” sounds much like the EQ-5D description “I am unable to perform my usual activities.” Although being “prevented” from doing something is not the same as being “unable” to do something, it is not clear that all participants made that distinction. Thus using SIDECAR-D in combination with EQ-5D could result in double-counting of quality of life. Nevertheless, double-counting remains a complex issue and depends on how a decision maker chooses to integrate the measures. In addition, SIDECAR-D items were each effectively valued in isolation. Hence, it may be that if a combination of several items were valued as a profile that valuations would differ.

The respondent sample is reasonably representative of the UK population, and there is no evidence that removing some responses from the analysis systematically excludes any particular section of society. Nevertheless, certain groups, in particular ethnic minorities, are not present in large numbers, and thus results may not accurately reflect their views.

A concern about using a general population sample might be that they were more familiar with EQ-5D items valuing general health than with carer-specific SIDECAR-D items. It is true that non-carers may value SIDECAR-D items differently than carers who have experienced them. Nevertheless, this is a well-known phenomenon, and is not limited to this instrument.^48^ In addition, very few respondents will have had personal experience of the EQ-5D level 3 descriptions. SIDECAR-D items are written in plain language, and reflect the words of ordinary carers. Thus they should be clear even to those without direct experience of caring, lowering the probability that general population respondents neglected them owing to unfamiliarity. The data bear this out: SIDECAR-D items were ascribed meaningful utility decrements.

There is much potential for future research building on this study. The 2 SIDECAR-D scoring systems (relative scale and tariff) could be used to evaluate services and interventions targeted at carers for people with dementia. Another potential use is evaluating services and interventions targeted at people with dementia themselves. For example, enabling people with dementia to be more independent can impact the burden of caring, thus improving carer quality of life. Existing tools and methods can struggle to capture such spillovers,^8^ thus SIDECAR-D may be a useful instrument to measure the wider impact of interventions.

The usefulness of SIDECAR-D in evaluation is an empirical question requiring further research. It will be important in the future to examine the validity of SIDECAR-D for ability to discriminate between groups of carers with established differences in quality of life. Other issues are how SIDECAR-D performs in practice in feasibility and whether interventions are capable of changing carer responses. It should also be investigated whether SIDECAR-D captures quality of life sufficiently alone, or whether specific health-related instruments (eg, EQ-5D) should be administered as well. Finally, future work should address to what extent SIDECAR-D captures spillovers, and to what extent the interventions that it is used to evaluate tend to be cost-effective.

Future research could address the validity of using SIDECAR to evaluate services/interventions for non-dementia carers, although SIDECAR was designed for carers for people with dementia, and at present is intended for use in this population only. The participants whose voices were used in the development of the measure were carers for people with dementia, and thus their concerns and priorities may not be the same as for other carers. Nevertheless, only 1 item refers specifically to dementia (“almost all of my conversations are about dementia or caring”), meaning it would be simple to adapt for other carer populations.

Finally, this study only assigns values to SIDECAR-D, and the development of SIDECAR demonstrated that the indirect impact of caring, and support and informational needs are captured by SIDECAR-I and SIDECAR-S. Future studies could value the other 2 instruments to help capture the full burden of caring for someone with dementia.

## Conclusion

This study presents an exercise valuing an instrument assessing the impact on quality of life of people who care for someone with dementia. Two scoring systems are the result, the SIDECAR-D relative scale measuring the relative impact on a scale from 0 to 100, and the SIDECAR-D tariff, measuring the impact on a scale with full health = 1 and dead = 0.
