# Supplementary material for: UK General Population Utility Values for the SIDECAR-D Instrument Measuring the Impact of Caring for People With Dementia
Source: Value Health. 2020 Aug;23(8):1079–86. doi: 10.1016/j.jval.2020.04.1827 (PMC7456787; doi:10.1016/j.jval.2020.04.1827)
Supplement: Supplemental Materials [file mmc1.docx]

You are being invited to participate in a research study titled ‘DECIDE’. This survey is being carried out by Professor Paul Kind and researchers from the University of Leeds.

This survey will take you approximately 20 minutes to complete.

The survey requires you to reflect on your experience of health including pain and illness which for some people may raise topics considered to be sensitive, embarrassing or otherwise upsetting. Previous experience suggests that although the topic may be distressing, participants find it a positive experience to express their views in a confidential environment.

Your participation in this study is entirely voluntary and you can withdraw at any time, however please be advised that any data you have provided up to that point will be kept. All data will be anonymised. Please be advised that due to the anonymous nature of this survey we will be unable to return any data to you if requested to do so.

We believe there are no known risks associated with this research study; however, as with any online related activity the risk of a breach is always possible. To the best of our ability your participation in this study will remain confidential, and only anonymised data will be published. We will minimise any risks by holding electronic data on a secure server at the University of Leeds and will comply with all aspects of the 2018 General Data Protection Regulation with data destroyed after 10 years. Ethical approval for this study has been sought from the School of Medicine Research Ethics Committee (ref MREC17-073).

**There are no right or wrong answers, we are just interested in what you think.**

A little bit about **you**

It will help us to understand the information you give us if you also answer a few general background questions about you .

**Q1** Are you … Male 🞏 Female🞏 Other/Prefer not to say 🞏

**Q2** What is your age in years?

*(please write in the box, values 1-100)*

A carer is anyone who cares, unpaid, (but perhaps receiving related social welfare beneifts) for a friend or family member who due to illness, disability, a mental health problem or an addiction cannot cope without their support

Q3 Are you a carer?

Yes 🞏 [go to Q4] No 🞏 [skip Q4, Q17-Q26]

Some information about the person that you provide care for

**Q4** What type of illness/condition is experienced by the person you care for?

| Asthma or other respiratory disorder |
| --- |
| Chronic fatigue syndrome/ME |
| Chronic kidney or liver disease |
| Chronic pain disorders (e.g. migraine or back pain |
| Heart disease or other circulatory disorder |
| Dementia |
| Depression |
| Diabetes |
| Epilepsy |
| Hepatitis |
| HIV |
| Gastrointestinal disease |
| Multiple sclerosis |
| Osteoarthritis |
| Parkinson’s disease |
|  |

Other illness or more detail………………………………………………

Some questions about **YOU**

**Q5** Please select a statement in each group below, that best describes your own health today

**Mobility**

I have no problems in walking about 🞏

I have some problems in walking about 🞏

I am confined to bed 🞏

**Self-Care**

I have no problems with self-care 🞏

I have some problems washing or dressing myself 🞏

I am unable to wash or dress myself 🞏

**Usual Activities**

(e.g. work, study, housework, family or leisure activities)

I have no problems with performing my usual activities 🞏

I have some problems with performing my usual activities 🞏

I am unable to perform my usual activities 🞏

**Pain/Discomfort**

I have no pain or discomfort 🞏

I have moderate pain or discomfort 🞏

I have extreme pain or discomfort 🞏

**Anxiety/Depression**

I am not anxious or depressed 🞏

I am moderately anxious or depressed 🞏

I am extremely anxious or depressed 🞏

Q6. Is there anything else which affects your health today?

………………………………………………………….

**Q.7** We would like to know how you think about your health **TODAY**.

On the next screen we will show you a scale from 0 to 100, with 0 being the **worst imaginable** health and 100 being the **best imaginable** health.


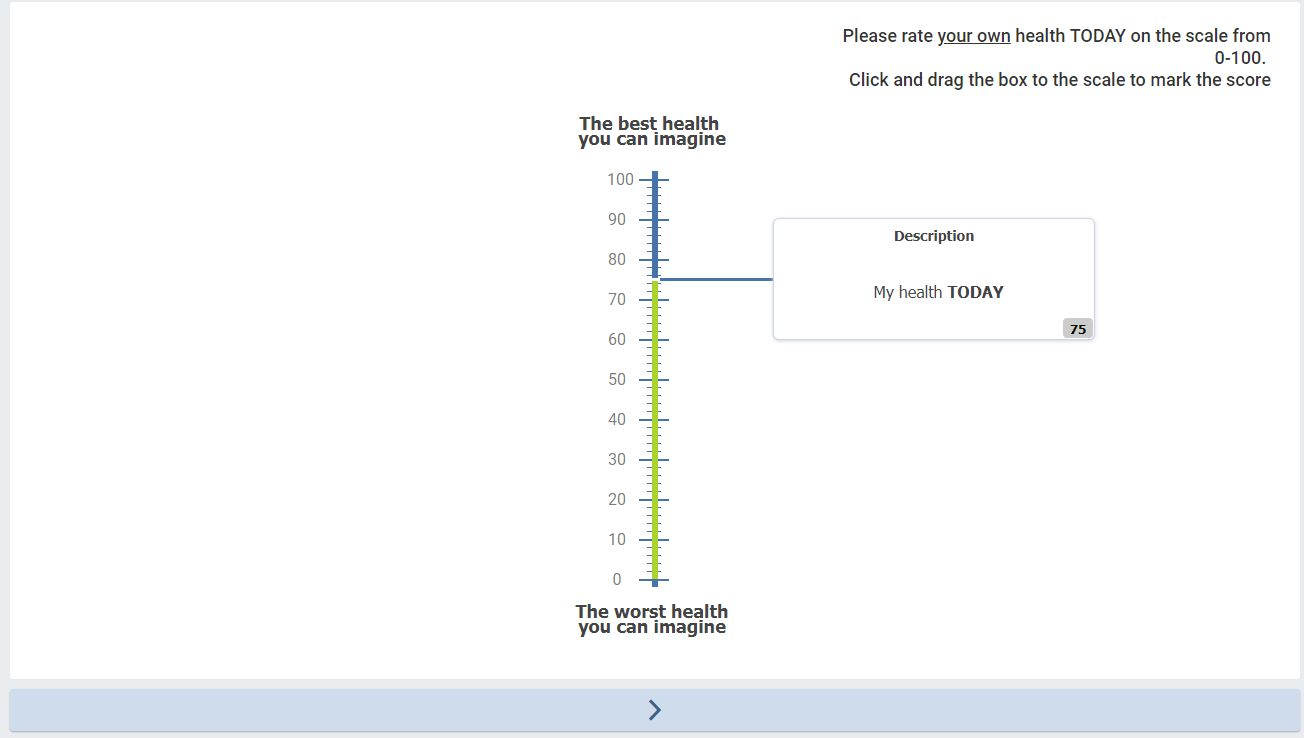


Q8. Do you have any long-standing illness, disability or infirmity?

### Yes 🞏 No 🞏

[If yes]

Please confirm what long-standing illness, disability or infirmity you have.

##### Q9.

In general, how would you describe your health?

*Please select a statement*

Excellent 🞏 Very Good 🞏 Good 🞏 Fair 🞏 Poor 🞏

*For each of the following questions, please select a score on the scale that you feel is most appropriate in describing you.*

##### Q10.

| - **Overall, how satisfied are you with your life nowadays?** | | | | | | | | | | | | | | | | | |
| --- | --- | --- | --- | --- | --- | --- | --- | --- | --- | --- | --- | --- | --- | --- | --- | --- | --- |
| 0 | 1 | | 2 | | 3 | 4 | | 5 | | 6 | | 7 | 8 | | 9 | | 10 |
| Extremely dissatisfied | |  | |  | | |  | |  | |  | | |  | | Extremely satisfied | |
| Q11. | | | | | | | | | | | | | | | | | |
| - **Overall, how happy did you feel yesterday?** | | | | | | | | | | | | | | | | | |
| 0 | 1 | | 2 | | 3 | 4 | | 5 | | 6 | | 7 | 8 | | 9 | | 10 |
| Extremely unhappy | |  | |  | | |  | |  | |  | | |  | | Extremely happy | |

### A bit more about you

| Q12. Which of the following best describes your situation? | |  |
| --- | --- | --- |
| *Please select a statement* | |  |
| Employed or self-employed | 🞏 | |
| Retired | 🞏 | |
| Housework | 🞏 | |
| Student | 🞏 | |
| Seeking work | 🞏 | |
| Prefer not to say | 🞏 | |
| Other (please specify) ­­­­­­­_______________________________ | | |

Q13. Did your education continue after the minimum school leaving age?

Yes 🞏 No 🞏

Q14. Do you have a degree or equivalent professional qualification?

Yes 🞏 No 🞏

**Q15.** Do you have any children under the age of 18, living with you as part of your family?

Yes 🞏 No 🞏

If yes, how many? ………………children

Q16. What ethnic group do you belong to?

| **White** | | |  | **Mixed/Multiple ethnic groups** |  |
| --- | --- | --- | --- | --- | --- |
| British | | | 🞏 | White and Black Caribbean | 🞏 |
| Irish | | | 🞏 | White and Black African | 🞏 |
| Gypsy or Irish Traveller | | | 🞏 | White and Asian | 🞏 |
| Other white background  Please describe ……………….. | | | 🞏 | Any other Mixed/Multiple ethnic background please describe……………………… | 🞏 |
| **Asian/Asian British** | |  | | **Black/** **African/Caribbean/Black British** |  |
| Indian | | | 🞏 | African | 🞏 |
| Pakistani | | | 🞏 | Caribbean | 🞏 |
| Bangladeshi | | | 🞏 | Any other Black/African/Caribbean background, please describe………………… | 🞏 |
| Chinese | | | 🞏 | **Other ethnic group** |  |
| Any other Asian background, please describe………………………….. | | | 🞏 | Arab | 🞏 |
|  |  |  |  | Any other ethnic group, please describe  **…………………………….**  **Prefer not to say** | 🞏  🞏 |
|  |  |  |  |  |  |

[Carer Specific Questions]

**Q17.** How long have you been a carer?

….....years …….months

**Q18**. How old is the person you care for?

……years

**Q19**. Is the person you care for male/female?

Male 🞏 Female 🞏

**Q20.** How many hours a day do you look after the person you care for?

Q21. About how long can you leave the person you care for at home alone?

| Never | **🞏** |
| --- | --- |
| Up to one hour | **🞏** |
| Up to half a day | **🞏** |
| A whole day | **🞏** |
| A whole night | **🞏** |
| A whole day and night, or longer | **🞏** |

**Q22.** What is your relationship to the person you care for?

| Partner | 🞏 |  | |  |  |  |  | |  |
| --- | --- | --- | --- | --- | --- | --- | --- | --- | --- |
| They are my parent | 🞏 |  | |  |  |  |  |  |  |
| I am their child | 🞏 |  | |  |  |  |  |  |  |
| Friend | 🞏 |  | |  |  |  |  |  |  |
| Other relative | 🞏 |  | (Please detail)  ………..…………............ | | | | |  |  |
| Other | 🞏 |  | (Please detail)  ……….………………… | | | | |  |  |

Q23. In general, how would you describe the health of the person you care for?

| Excellent 🞏 | Very Good 🞏 | Good 🞏 | Fair 🞏 | Poor 🞏 |
| --- | --- | --- | --- | --- |

Q24. How would you describe the severity of the person’s dementia? [Only for dementia carers.]

| Mild - Needs some assistance with day-to-day life due to dementia but is still quite independent | **🞏** |
| --- | --- |
| Moderate – Has obvious difficulties with memory or thinking due to dementia and needs a lot of assistance with day-to-day life | **🞏** |
| Severe – Has great difficulty communicating and needs help with many aspects of personal care (e.g. washing, getting dressed and eating) | **🞏** |

Q25. **Thinking about the person you care for,** please select a statement in each group below, best describes **their** health **today**.

| **Mobility** |  |
| --- | --- |
| They have no problems in walking about | 🞏 |
| They have some problems walking about | 🞏 |
| They are confined to bed | 🞏 |
| **Self-Care** |  |
| They have no problems with self-care | 🞏 |
| They have some problems washing or dressing himself/herself | 🞏 |
| They are unable to wash or dress himself/herself | 🞏 |
| **Usual Activities** |  |
| (e.g. work, study, housework, family or leisure activities) |  |
| They have no problems with performing usual activities | 🞏 |
| They have some problems with performing usual activities | 🞏 |
| They are unable to perform usual activities | 🞏 |
| **Pain/Discomfort** |  |
| They have no pain or discomfort | 🞏 |
| They have moderate pain or discomfort | 🞏 |
| They have extreme pain or discomfort | 🞏 |
| **Anxiety/Depression** |  |
| They are not anxious or depressed | 🞏 |
| They are moderately anxious or depressed | 🞏 |
| They are extremely anxious or depressed | 🞏 |

**Q26.** Now, we would like you to think about the health of the person you care for **TODAY**.

On the next screen we will show you a scale from 0 to 100, with 0 being the **worst imaginable** health and 100 being the **best imaginable** health.

Please indicate how you’d rate the health of the person you care for.


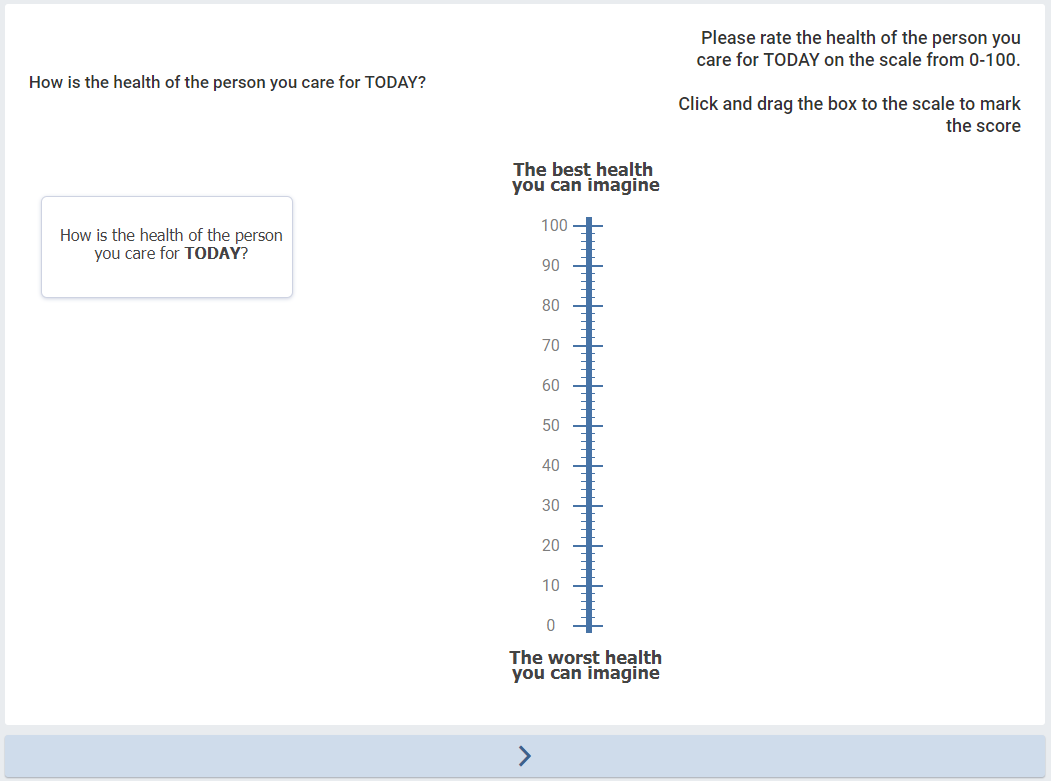


**Best/Worst Scaling**

*In each question in the next part of the survey, we are going to show you a list of statements.*

*The statements relate to aspects of caring and health which have a negative impact on people’s lives.*

*Most statements appear in more than one question, but the list of statements is different for each question.*

*A carer is anyone who cares, unpaid, for a friend or family member who due to illness, disability, a mental health problem or an addiction cannot cope without their support.*

*If you are not a carer, please imagine that you are when answering the questions.*

*Here is an example question:*

| MOST NEGATIVE impact  (choose ONE) |  | LEAST NEGATIVE impact  (choose ONE) |
| --- | --- | --- |
| q | It is hard to find anyone else to spend time looking after the person I care for | q |
| q | I don't take very good care of myself | q |
| q | Little things add up to make caring difficult | q |
| q | I would like it if others tried harder to understand the situation I am in | q |
| q | I am moderately anxious or depressed | q |
| q | I have some problems washing or dressing myself | q |

*In the left-hand column, you should choose the ONE statement you think would have the MOST NEGATIVE impact on your quality of life.*

*In the right-hand column, you should choose the ONE statement you think would have the LEAST NEGATIVE impact on your quality of life.*

*Just because a statement has the least impact on quality of life doesn’t mean it has no impact.*

*You may think that all the statements would have a big impact on your quality of life.*

*In this case, you should still choose the one which has the least impact, i.e. you think all other statements have an even more negative impact.*

*We are interested in your own personal opinions.*

*There are no right or wrong answers.*

*There are EIGHT questions in this part of the survey.*

**BWS Question 1**

*Consider the statements below.*

*In the column on the left, select the ONE statement which would have the MOST NEGATIVE impact on your quality of life.*

*In the column on the right, select the ONE statement that would have the LEAST NEGATIVE impact.*

| MOST NEGATIVE impact  (choose ONE) |  | LEAST NEGATIVE impact  (choose ONE) |
| --- | --- | --- |
| q | I regularly have to do things as a carer that I am not comfortable with | q |
| q | I can only get through one day at a time | q |
| q | Caring prevents me from fulfilling my other responsibilities, (e.g. working, being a parent, volunteering) | q |
| q | Little things add up to make caring difficult | q |
| q | I have some problems with performing my usual activities | q |
| q | There is always something new to deal with when providing care | q |

[7 more BWS questions follow]

**Q27.** Some people think that there are health conditions worse than being dead. This is a very sensitive issue, and people have different opinions.

On the next screen we will show you three descriptions.

Please rate each one on a scale from 0 to 100, with 0 being the **worst imaginable** health and 100 being the **best imaginable** health.

There are no right or wrong answers; we would like to know your opinion.


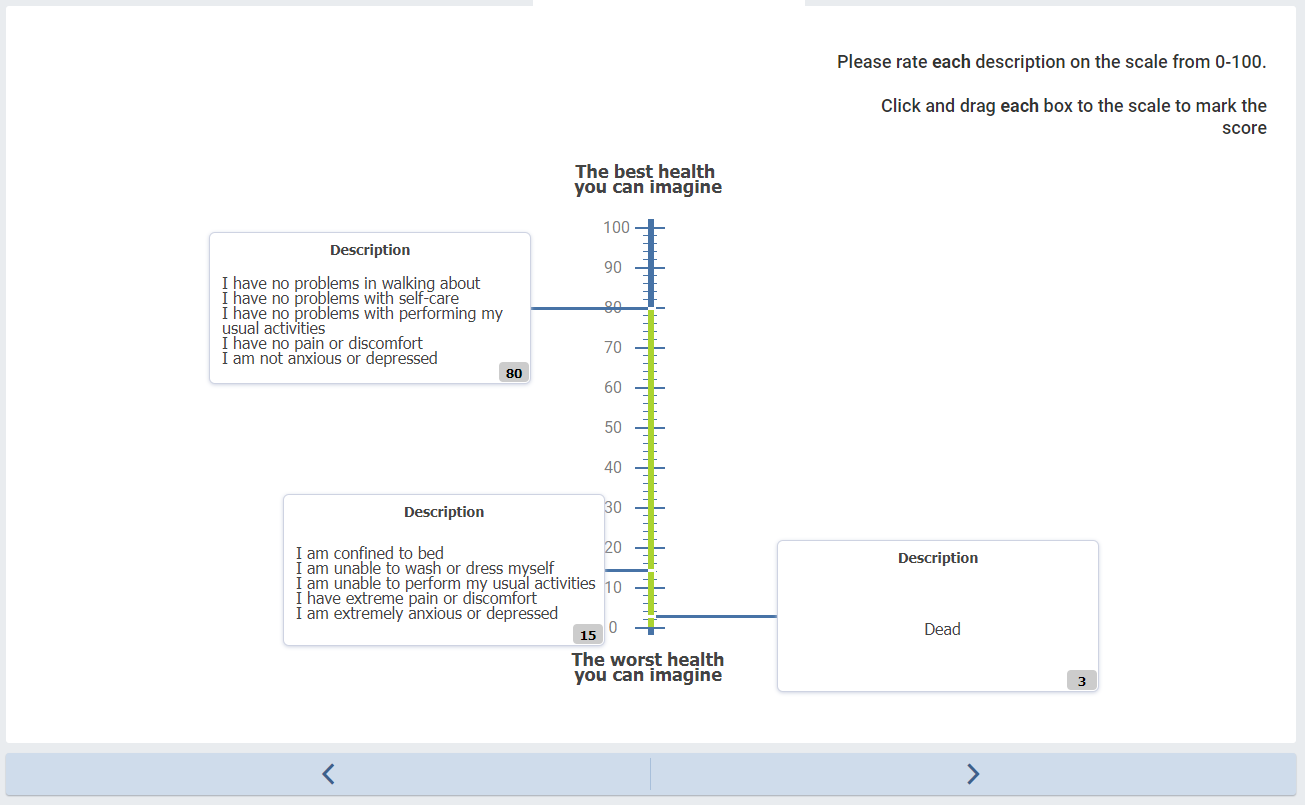


**Q28.** How confident are you that you understood the final health rating question? (SC)

| Very Confident | Somewhat confident | Undecided | Not really confident | Not confident at all |
| --- | --- | --- | --- | --- |
| **🞏** | **🞏** | **🞏** | **🞏** | **🞏** |

**[Carers then completed the SIDECAR-A instrument for themselves. To obtain the SIDECAR-A instrument, please register by following the link at** [**http://decideproject.co.uk/**](http://decideproject.co.uk/)**]**

Thank you for your time
